# Draw-Care, a Co-Designed Multilingual Digital Intervention for Family Carers of People Living With Dementia From Ethnically Diverse Communities: User-Testing Study

**DOI:** 10.2196/81128

**Published:** 2026-03-03

**Authors:** Antonia Thodis, Thu Ha Dang, Nilmini Wickramasinghe, Nalika Ulapane, Claudia Cooper, Briony Dow, Duncan Stuart Mortimer, Tuan Anh Nguyen, Lily Dongxia Xiao, Josefine Antoniades, Andrew Simon Gilbert, Joanne Enticott, Mary Gurgone, Mathew Varghese, Santosh Loganathan, Bianca Brijnath

**Affiliations:** 1 The George Institute for Global Health Sydney, New South Wales Australia; 2 School of Health Sciences Swinburne University of Technology Hawthorne, Victoria Australia; 3 School of Computing, Engineering and Mathematical Sciences La Trobe University Bundoora, Victoria Australia; 4 Wolfson Institute of Population Health, Queen Mary University of London London United Kingdom; 5 National Ageing Research Institute Parkville, Victoria Australia; 6 Centre for Health Economics Monash University Melbourne, Victoria Australia; 7 College of Nursing and Health Sciences, Flinders University Adelaide, South Australia Australia; 8 School of Humanities and Social Sciences La Trobe University Bundoora, Victoria Australia; 9 Monash Centre for Health Research and Implementation Monash University Clayton, Victoria Australia; 10 Association for Culturally Appropriate Services Perth, Western Australia Australia; 11 St John’s Medical College Bengaluru, Karnataka India; 12 National Institute of Mental Health and Neurosciences Bengaluru, Karnataka India

**Keywords:** carer, co-design, dementia, digital intervention, ethnically diverse

## Abstract

**Background:**

Technology can deliver culturally and linguistically appropriate resources to support ethnically diverse family carers (hereafter referred to as carers) of people living with dementia. However, carers’ involvement in research on the development and evaluation of such digital health interventions is limited.

**Objective:**

This study aims to user-test the co-designed Draw-Care multilingual, web-based dementia resource with and for carers.

**Methods:**

We evaluated the web-based resource through observation sessions to collect carer feedback, using a mixed methods approach. This comprised the online, validated eHealth Literacy Scale and survey questions assessing the perceived usefulness and importance of the internet for health-related decision-making and access to health resources. In addition, “think-aloud” website navigation sessions were conducted, and Hotjar analytics were used to capture participants’ behavior on the website. Quantitative and analytics data were analyzed descriptively, and qualitative data were analyzed using instant data analysis, followed by thematic analysis.

**Results:**

Between March and April 2023, a total of 30 carers participated in the user-testing sessions (women, n=20, 67%; mean age 61, SD 13.5 years). The mean eHealth Literacy Scale score was 30 (SD 6.1). Overall, 18 (60%) participants perceived the internet as useful, and laptops and tablets were the most commonly used devices for accessing resources, each used by 9 (30%) participants. Vietnamese (n=5, 17%), Mandarin (n=5, 17%), and English (n=5, 17%) were the top 3 languages the resource was accessed in. A total of 28 (93%) participants could navigate and log in to the website with little to no support. Qualitative results showed that overall, the Draw-Care web-based resource was acceptable, culturally responsive, engaging, and usable. However, navigation was more complicated for those using smaller screens (eg, smartphones and tablets). There were linguistic discrepancies arising from translation issues in Vietnamese and Mandarin, and 14 (46%) participants found it difficult to identify and use the chatbot (ie, virtual helper interface). Issues identified with the prototype Draw-Care website and the proposed improvements included eliminating the virtual helper, simplifying the rating scale from a 5-point smiling emoticon scale to a 3-star rating scale, improving the visibility of the feedback button, and ensuring translation accuracy.

**Conclusions:**

To our knowledge, this is the first study to evaluate a bespoke multilingual website delivering a novel, co-designed, and culturally adapted digital intervention in 10 languages for ethnically diverse family carers of people living with dementia. Findings from this user-testing study undertaken with carers uncovered usability issues requiring remediation and emphasized the importance of inclusive, accessible, culturally sensitive, engaging, and beneficial content and design. Key revisions were implemented before the randomized controlled trial commenced.

**International Registered Report Identifier (IRRID):**

RR2-10.1177/20552076231205733

## Introduction

Ethnically diverse communities experience a disproportionately high and growing burden of dementia [1-3]; however, they continue to face substantial barriers to accessing timely, culturally and linguistically appropriate support [4,5]. Family members of people with dementia from ethnically diverse backgrounds often provide care alone, which adversely affects their health and economic well-being [6]. They report limited awareness of available services, difficulty navigating complex care systems, language barriers, culturally insensitive service models, and a lack of translated or tailored information [5,7-9]. These unmet needs contribute to higher psychological distress, greater social isolation, and reduced care continuity for ethnically diverse carers and people living with dementia [10,11].

Digital technologies can partially address some of these inequities by providing flexible and accessible support that improves carers’ psychological health, self-efficacy, caregiving skills, and problem-solving abilities [11]. The World Health Organization’s iSupport program is one such digital intervention [12]; although it has been adapted in more than 40 countries, no comparable work has been completed for iSupport Lite (a simplified version of iSupport that includes 6 visual messages to support carers) [13]. Research to include ethnically diverse family carers (hereafter referred to as carers) of people living with dementia in the development and testing of culturally responsive digital media resources remains limited [14,15]. Existing adaptations often require substantial localization to reflect cultural norms, family structures, and beliefs about dementia. In addition, many digital resources for culturally and linguistically diverse carers provide only translated information without meaningful cultural tailoring, which may contribute to low uptake and engagement [16-18]. To advance health equity through technology [19], further research is needed to explore technology’s potential to bridge gaps in caregiving provision [15,19,20].

Addressing these gaps, the Draw-Care study culturally adapted the iSupport Lite dementia carer online support messages with carers and service providers and co-designed a multilingual, culturally responsive website [21]. The co-designed prototype intervention comprised 6 short, animated films and tips sheets hosted on the website, along with a virtual helper, delivered in 10 languages: Arabic, Cantonese, Greek, Hindi, Italian, Mandarin, Spanish, Tamil, Vietnamese, and English [17].

Before testing the intervention’s clinical and economic effectiveness in a parallel, 12-week waitlist randomized controlled trial (RCT), user testing was undertaken to refine the intervention with ethnically diverse carers to examine its usability and acceptability. This paper reports the findings from the user-testing component. The specific study aims were to assess the website’s usability, accessibility, and ease of navigation across the aforementioned 10 languages and to identify any refinements required before RCT implementation to test the intervention. The specific research questions guiding the user testing were as follows: how usable, accessible, and easy to navigate is the website for ethnically diverse carers from the aforementioned linguistic backgrounds? What modifications, if any, are needed to optimize the website for broader implementation and rigorous testing in a future RCT?

## Methods

### Study Setting and Participants

Before the RCT, the Draw-Care study comprised the co-design and development of the Draw-Care digital intervention, including member checking and user testing of the web-based resource. Between March and April 2023, a total of 65 ethnically diverse carers from our existing networks and previous studies [22], across all 10 language groups, were invited to evaluate the co-designed Draw-Care intervention. Member checking was undertaken with participants from the co-design phase (ie, health care providers and current carers) to ensure that the prototype reflected the feedback gathered during the co-design of the resources. Details of this have been published elsewhere [21].

User-testing sessions with the intended end users were subsequently conducted as part of the co-design, development, and refinement of the intervention before the trial. Unlike the member-checking step, eligibility criteria for participants in the user-testing sessions included being a current or former carer of a person living with dementia; aged >18 years; not having participated in the co-design of Draw-Care; having access to the internet and a device (eg, computer, phone, or tablet); and being from an Arabic-, Cantonese-, Greek-, Hindi-, Italian-, Mandarin-, Spanish-, Tamil-, or Vietnamese-speaking background. These 9 languages are among the top 10 non-English languages spoken by people aged >65 years in Australia, and English is the official language of Australia [21]. The sample size for online user-testing sessions was estimated to be between 27 to 45 eligible participants, based on enrolling 3 to 5 carers for each language group (excluding English).

### Ethical Considerations

The Draw-Care study was approved by the Human Research Ethics Committee of Curtin University, Australia (HRE2022-0004) and the National Ageing Research Institute Research Governance Office. The trial was prospectively registered on the Australian New Zealand Clinical Trials Registry (trial registration numbers ACTRN12622000382774 and ACTRN12622000358741). All participants provided written informed consent and received a US $30 gift card for their time. Participants were able to opt out of the study prior to their data being de-identified, aggregated, and analysed.

### Procedure

Using REDCap (Research Electronic Data Capture) [23], all participants completed a sociodemographic questionnaire. The questionnaire included a question to self-report their English proficiency level with the following response options: none or no proficiency, basic or simple proficiency, conversational proficiency (ie, can carry a conversation but is not fluent), professional or high-level proficiency (but not at the level of a native speaker), and native speaker (highest level of fluency and proficiency).

Participants also completed the 8-item eHealth Literacy Scale (eHEALS) [24] and questions on the perceived usefulness and importance of the internet for health-related decision-making and access to health resources (Multimedia Appendix 1). Although the psychometric properties of the validated eHEALS can vary across populations and translations, there is agreement in scientific literature that it is a reliable and robust measure of perceived eHealth literacy, demonstrating strong internal consistency (measured by Cronbach α with values ranging from 0.84 to 0.97) and significant correlations with related measures that support its validity [25,26]. Designed to measure an individual’s confidence in their ability to locate and evaluate online health information, eHEALS has been widely used and tested across diverse populations [27,28].

Subsequently, via an online “think-aloud” interview, the researchers (AT and THD) conducted a user-testing session with each participant. A log-in code was emailed to each participant to access the website. Codes were activated when the session started and remained active after capturing user behavior analytics using embedded Hotjar (Contentsquare) [29]. Each session lasted 20 to 30 minutes. The researchers asked participants to complete sequential, representative website tasks and provide feedback while “thinking-aloud” and screen sharing (Multimedia Appendix 2). Participants were asked to select their preferred language for navigating the site, log in to the website using the code provided, follow the website prompts to the home page titled “Films and More Information,” and then commence the sequential tasks with the researcher (Multimedia Appendices 2 and 3). All participants completed each component. Sessions were not recorded; therefore, the researchers took extensive notes.

### Analysis

Survey data were analyzed descriptively using SPSS (version 28.0; IBM Corp) [30]. The sample size limited subgroup analyses by language group; therefore, these analyses were not conducted. An eHEALS score was calculated for each participant, and the aggregated mean score was reported. Higher scores (range 8-40) indicate greater self-perceived skills in finding, evaluating, and using electronic information to make decisions about individual health.

Issues, frustrations, and comments made by the participants during the “think-aloud” sessions were recorded by AT or THD in an Excel (Microsoft) spreadsheet and analyzed to identify and rank website usability issues using instant data analysis (IDA) [31]. At the end of each interview, they listed the usability issues observed. The issues were ranked as critical (participant unable to complete the task), severe (significant delay or frustration in completing the task), or cosmetic (minor issues). Each issue was annotated with precise references to the website, alongside supplementary notes detailing the nature of the problem and participants’ reactions [31]. This detailed annotation process helped identify when data saturation, (ie, no new usability issues) had occurred.

After all the sessions were completed, the identified usability issues were aggregated into broader themes using a thematic analysis approach [32]. As we assumed that participants’ accounts reflected and were mediated through their real-world experiences, a postpositivist thematic analysis was conducted to analyze the entire dataset and identify key themes through a comparative and iterative process [32]. During this stage, AT and THD independently coded the data, met to create consensus, resolved discrepancies, and developed themes. BB checked the final codes and themes and moderated where there were conflicts. Attention was paid to comparing findings across linguistic groups. Thus, the use of multiple coders and consensus-based coding ensured data triangulation and robust interpretation, enhanced thematic and coding reliability, and minimized researcher bias [32]. Combining IDA and thematic analysis generated a consolidated list of usability issues and proposed modifications to overcome them, an approach successfully applied in previous digital health intervention studies [33,34].

**Figure 1:**
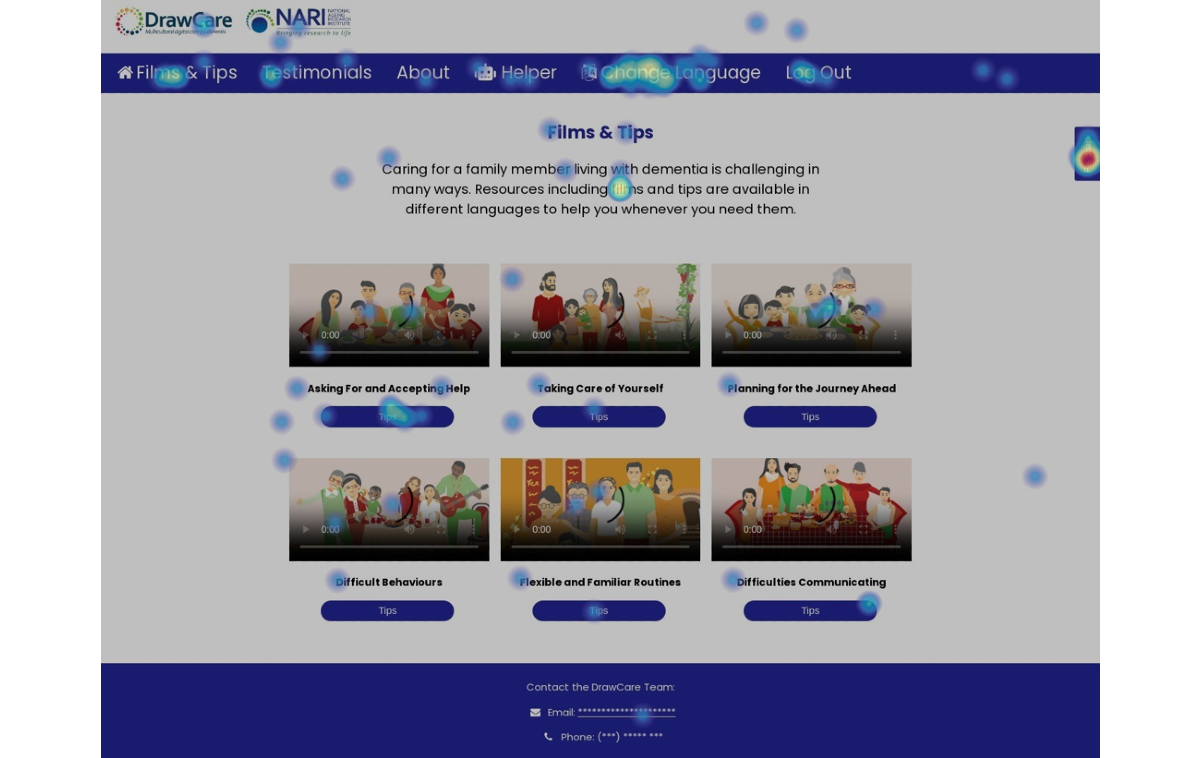
Screenshot of the Draw-Care website’s “Films and Tips” home page showing the location of the feedback button (top right-hand side) covered by a red heat map.

**Figure 2:**
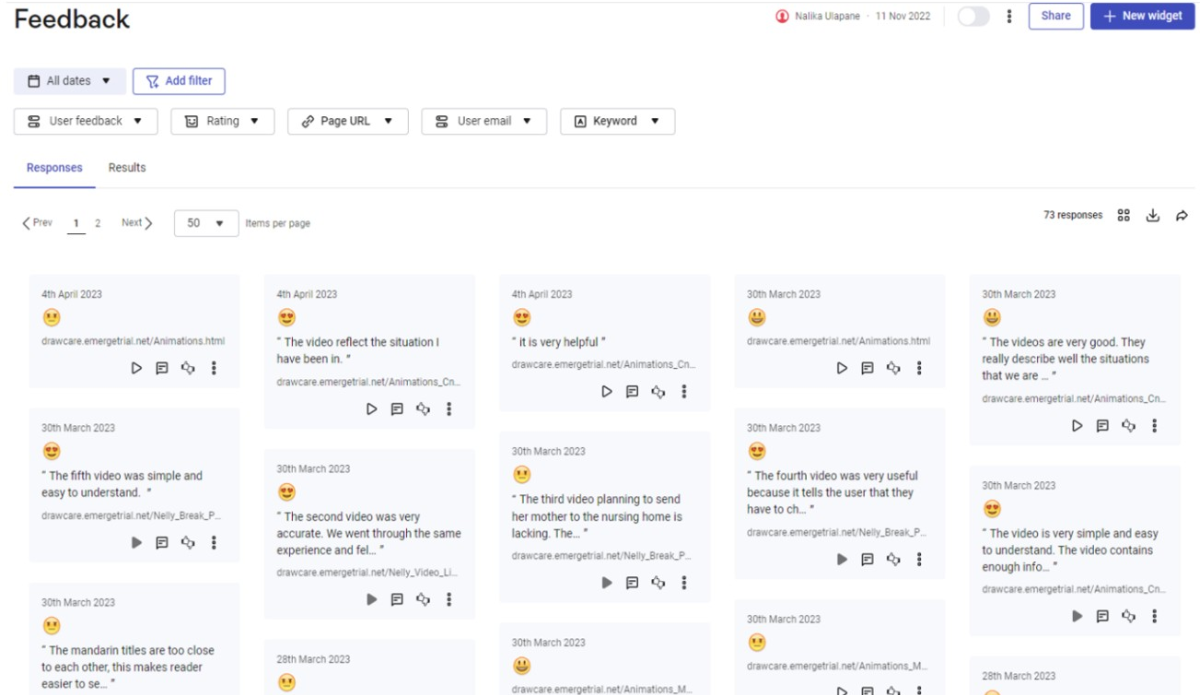
A sample of the feedback and film ratings captured from participants who used the feedback feature and the 5-point emoticon rating scale on the Draw-Care website.

**Figure 3:**
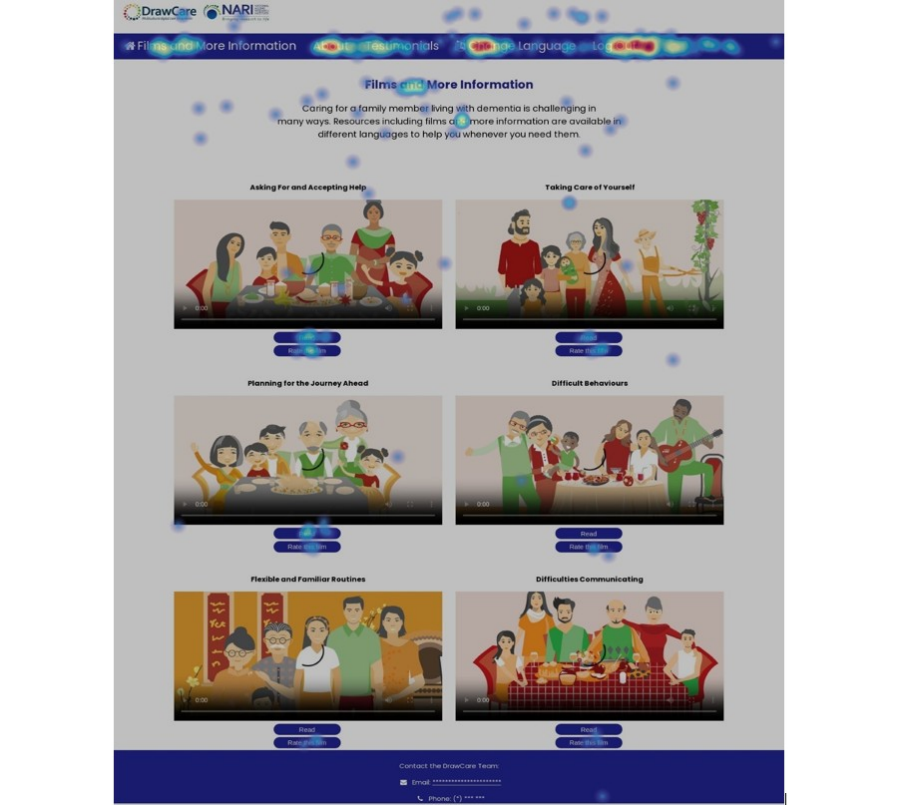
Screenshot of the Draw-Care website’s “Films and Tips” home page with a heat map recording showing user movements by color coding (blue=less activity and red=more activity).

Analytics data were aggregated to generate visual heat maps of the overall website, individual pages, and elements of a page that participants did or did not engage with. Screenshots of the website’s feedback feature and samples of user feedback and visual heat maps are shown in Figures 1, 2, and 3, respectively.

## Results

### Participant Characteristics

A total of 30 carers participated in the study (with a 46% response rate), comprising mainly women (n=20, 67%). Participants’ mean age was 61 (SD 13.5) years, and they were related to the person living with dementia as a daughter (n=15, 50%), wife (n=4, 13%), daughter-in-law (n=3, 10%), son (n=3, 10%), husband (n=2, 6%), or granddaughter (n=1, 3%). Mean duration of caregiving was 7.1 (SD 3.5) years. Most participants were born overseas (n=22, 73%), and the mean duration of residence in Australia was 43 (SD 11.7) years. All participants were either Australian citizens (n=26, 87%) or permanent residents (n=4, 13%). A total of 26 (87%) participants spoke a language other than English at home, including Chinese (n=6, 20%), Vietnamese (n=5, 17%), Hindi (n=5, 17%), Greek (n=5, 17%), Italian (n=3, 10%), Arabic (n=1, 3%) and Spanish (n=1, 3%). Few only spoke English at home (n=2, 7%) and data was missing for 2 (7%) participants (Table 1).

From the IDA and thematic analysis, website issues were inductively analyzed into two themes: (1) acceptability and (2) usability. Table 2 describes how the themes, codes, and IDA findings were grouped. Website issues are detailed and supported by illustrative quotes, labeled with deidentified participant ID number, age (years), gender, and preferred language. Where relevant, descriptive quantitative data are integrated to provide further context.

**Table 1:** Sociodemographic characteristics of the participants (N=30).

Characteristics			Participants, n (%)
**Marital status**			
	Married		20 (67)
	Single^a^		10 (33)
**Living circumstances**			
	Lives with others		19 (63)
	Lives alone		11 (37)
**Work status**			
	**Employed**		
		Full time	6 (20)
		Part time	3 (10)
		Casual	5 (17)
	Retired		14 (47)
	Unemployed		2 (7)
**Highest education level**			
	Primary		1 (3)
	Secondary		10 (33)
	Tertiary		19 (63)
**Self-reported English proficiency level^b^**			
	None		1 (3)
	Basic		6 (20)
	Conversational		5 (17)
	Professional		10 (33)
	Native		8 (27)

^a^Single includes the following response options: single, never married, and divorced or separated.

^b^Response options were adapted from the International English Language Testing System categories: none=no proficiency, basic=simple proficiency, conversational=can carry a conversation but not fluent, professional=high level of proficiency but not at native speaker level, and native=highest level of fluency and proficiency.

**Table 2:** Summary of thematic analysis and instant data analysis (IDA).

Theme and code			Issues identified in IDA
**Website acceptability**			
	Presentation	First impressions and overall look and feel of the website	
	Navigation	Ease of navigation varied depending on the device used	
	Understanding	Linguistic discrepancies (human translation)	
**Website usability**			
	Interface interaction	Chatbot interface (technology-related issues)	
	Satisfaction	Use of emoticons Cultural misunderstanding of emoticons	
	Accessibility	Monolingual feedback button Cultural misunderstanding of feedback request	

### Website Acceptability

Participants perceived themselves to be relatively adept at finding, evaluating, and using online health information, with the mean eHEALS score being 30 (SD 6.1). Most participants perceived the internet as *useful* (n=18, 60%) or *very useful* (n=6, 20%), and only 6 (20%) were unsure. Most participants rated the internet as *important* (*n*=13, 43.3%) or *very important* (*n*=12, 40%), with 3 (10%) unsure and 2 (7%) rating it as *not important* or *not important at all*.

Consistent with their high eHEALS score, a diverse array of devices was used to access the website, including laptops (n=9, 30%), tablets (n=9, 30%), PCs (n=7, 23%), and smartphones (n=5, 17%). The most common languages the website was accessed in were Vietnamese (n=5, 17%), Mandarin (n=5, 17%), English (n=5, 17%), Cantonese (n=4, 13%), Greek (n=4, 13%), Hindi (n=3, 10%), Italian (n=2, 7%), Spanish (n=1, 3%), and Arabic (n=1, 3%).

In terms of website navigation, 28 (93%) participants identified the log-in box, entered the code, and located the home page and menu bar (Multimedia Appendix 3). Most carers (n=25, 83%) located the films and tips sheets, watched an animation, and read the tips sheets without researcher’s instruction. Qualitative feedback regarding acceptability was positive. The presentation and first impressions indicated that a clear color branding was used, text size and fonts across all languages were clear and readable, and the film content and tips sheets were cohesive and informative:

Chinese writing, strokes, and characters are good in subtitles and on the website.

ID 21, woman, aged 60 years, Mandarin

Good, informative, and clear. The animation was consistent with the message [in the accompanying tips sheet].

ID 1, woman, aged 60 years, Spanish

However, locating the menu bar on the home page depended on the device used and screen size. On Mac laptops and smaller screens (eg, tablets and smartphones), the website layout changed, and some participants struggled to navigate it on these devices:

The horizontal menu becomes vertical with the three short bars on the top-right. Not everyone knows that they need to click on this bar to find the menu.

ID 7, woman, aged 63 years, Hindi

Linguistic translations of “tips” in Vietnamese and “dementia” in Mandarin were also found to be unacceptable. The intended use of “tips” was to denote a brief, useful piece of information accompanying each film’s theme, but in Vietnamese, the term meant a gratuity. The translation of “Dementia” to Mandarin was incorrect and outdated, with some terms overly technical or difficult to understand:

The video is very good and helpful. Just a suggestion...some of the medical terms in Chinese is difficult...e.g. the medical term [for] “Alzheimer”...

Deidentified user ID 49

### Website Usability

#### Overview

When prompted to locate and use the virtual helper to select 1 of the 6 films, 16 (53%) participants completed this task without further instruction. Most participants (n=20, 67%) correctly located and used the film rating scale. Heat map recordings were captured to visually track how users interacted with the website, with interaction intensity ranging from red=busy interactions to blue=fewer active interactions. An example of an aggregated heat map is shown in Figures 1 and 3.

Data were captured through user movements such as clicks or page scrolls. There were 199 aggregated user movements and heat map recordings, which on average suggested low user frustration, based on the low proportion of rage clicks, and high website engagement, based on movements patterns such as landing mostly on the films and tips page. User movements on average indicated about 28 minutes spent scrolling on this page, which is the core of the intervention. According to user feedback from the think-aloud sessions and heat map recordings, three features were unclear: (1) the virtual helper; (2) the 5-point emoticon or smiley face film rating scale; and (3) the feedback button. These features are discussed in the subsequent sections.

#### Virtual Helper

The “helper”—a simple, non–artificial intelligence chatbot—was denoted by a smiling robot icon intended to help users locate which short films addressed their needs. Clicking on the icon revealed a list of problematic caring scenarios that the films then addressed. A total of 14 (47%) of the 30 participants required instruction to locate and use the helper, as the icon did not prompt users to click on this function. On smaller screens, the icon was too small to view clearly and was difficult to locate on the menu bar. The word “helper,” when translated into other languages, was also unclear. For example, the Italian translation was perceived as a helper who assists in caring for a person living with dementia. Feedback to improve usability included changing the icon to a question mark symbol to indicate help-seeking and adding short multilingual “pop-up” explanations about its purpose.

#### Five-Point Emoticon Rating Scale

The 5-point emoticon rating scale used smiley faces ranging from a very happy face to a very sad face. Emoticons appeared after watching a film to capture an optional rating of likability and satisfaction of the film. However, the emoticons were culturally incongruent and frequently misinterpreted. Participants selected an emoticon that described their emotional state as a carer rather than a rating of satisfaction or dissatisfaction with the film itself. Feedback to improve usability included replacing emoticons with stars and reducing the options from 5 to 3:

[A] star icon is better than “emotion” icon because the emotion can be understood differently in different cultures.

ID 2, man, aged 62 years, Cantonese, and Mandarin

#### Feedback Button

The “feedback” button was designed to capture users’ optional, deidentified website feedback by easily clicking on the button to enable a gray pop-up text box with the phrase “Tell us about your experience” (Figure 2). Due to software limitations, it was not possible to translate this phrase, which remained in English, unlike the remaining website text. Consequently, some participants were unable to provide feedback because they could not type in English and in their preferred language. Participants who located the feedback button provided feedback that was overall positive (Figures 1 and 2). However, several carers misinterpreted the feedback request as referring to their caregiving experiences rather than their experience using the website:

In the “feedback,” it asked about the user’s experience. It is not clear for carers: their experience in using the website or in care work.

ID 3, woman, aged 57 years, Vietnamese

User testing further revealed that the feedback button’s appearance and position were also too small and hidden on devices with smaller screens. Repositioning the button as a discreet tab on the main menu bar was recommended to improve visibility and usability.

After collating the feedback, several changes were made to improve the website’s acceptability and usability. These revisions are detailed in Textbox 1.

**Acceptability and usability issues identified from user testing and proposed revisions to improve the Draw-Care intervention (before testing the intervention’s effectiveness in a randomized controlled trial [RCT]).**

**Key usability and acceptability issues identified from user testing of the prototype Draw-Care website**

- Ease of access to the nonpublic prototype website was hindered by the case sensitivity of the log-in code.
- Poor visibility of resources made the animations and tips sheets difficult to locate on smaller screens.
- Positioning of film titles under image windows was not user-friendly or intuitive, thus requiring users to scroll down the page.
- Virtual helper feature: the purpose and terminology translated from English to other languages lacked clarity, the feature was not easily located, and the robot icon that visually symbolizes “help” was not intuitively obvious.
- Feedback button and text box pop-up: the purpose, location, and size were unclear and only usable in English text.
- The function and purpose of the 5-point emoticon-based optional film rating scale were unclear and culturally incongruent.
- Linguistic discrepancies in key terms:
  Dementia (Mandarin only)
  Tips (all languages)

**Proposed revisions to improve the acceptability and usability of the prototype Draw-Care website (before commencement of the RCT)**

- Written instructions will be provided to RCT participants to streamline website access, noting the case sensitivity of the log-in code.
- Film titles will be repositioned above the animated images.
- A “Read” button will be added to direct users to information sheets.
- The color of the buttons for “Watch” and “Read” will change as users hover over these options to indicate that clicking on each will play the film or direct the user to the information sheet.
- Font size of the text will be increased, and text will be repositioned to improve optimization on screens of all devices.
- The virtual helper function will be removed; therefore, the term “helper” in all languages will be removed from all website text.
- The feedback button and text box pop-up will be removed.
- Emoticons will be replaced with an optional 3-star rating scale at the end of each film.
- Revisions and retranslations will be conducted where required, including the following:
  Replacing the outdated terminology of dementia with the correct version in all Mandarin resources.
  Changing the title “Tips” to “More Information” and consequently changing the title “Films and Tips” to “Films and More Information.”
- One round of review of revised resources will be conducted with selected users.

## Discussion

### Principal Findings

To our knowledge, this is the first study to evaluate a bespoke multilingual website that hosts a novel, co-designed, culturally adapted digital intervention in 10 languages, including English, for ethnically diverse family carers of a person living with dementia. On the basis of feedback, film ratings, and aggregated Hotjar data analytics, the website design, layout, and content, including films and information sheets, were culturally acceptable and engaging. The information was useful and aligned with the corresponding film messages; however, the purpose and positioning of the virtual helper, the film rating scale, and the feedback button were not intuitive. Linguistic discrepancies occurred despite an extensive co-design process and professional translation of the text and resources. For example, linguistic translations for “helper” and “tips” did not help users understand their function in all the languages, which created confusion. Similarly, the translation of dementia from English to Mandarin (ie, 痴呆症), while a clinical term, can also connote “stupidity.” During the user-testing phase (within 12 months of co-design and website development), this terminology was considered outdated, with emerging nomenclature favoring more neutral and less stigmatizing terms (ie, 失智症, meaning “loss of cognition”) [18].

Access to the nonpublic prototype website was occasionally hindered by the case-sensitive log-in code, which some participants found difficult to enter correctly. o streamline access in the RCT, written instructions were provided to all participants, explicitly highlighting the case sensitivity of the log-in code and offering clear, step-by-step guidance for logging in.

These findings underscore the importance of engaging end users to ensure digital interventions function as intended and are understood clearly before proceeding to the RCT and the rollout of a web-based intervention, particularly when the primary focus is on ensuring that resources are culturally responsive beyond language translation alone [35]. Such steps are necessary, albeit expensive and time consuming, even when the resources are co-designed with end users.

Study strengths include the use of a mixed methods approach, experienced bilingual researchers (with lived experience of dementia care), and rapid inductive analysis to revise the web-based resource before undertaking the RCT to test its effectiveness with carers from ethnically diverse backgrounds who had not participated in the co-design phase, which is likely to encourage unbiased feedback related to usability.

### Limitations

Despite achieving a sample size within the estimated range (n=27 to 45), not all 10 language groups targeted by the prototype intervention were represented (eg, Tamil-speaking carers). Most participants had a moderately high level of eHealth literacy, and all participants were within a category of Australian residence that enabled access to government-subsidized services. Results might have been different if participants had lower eHEALS scores and limited access to publicly subsidized services.

### Conclusions

The Draw-Care study is one of the first to investigate how to make digital interventions more ethnically inclusive, thereby better supporting ethnically diverse family carers of people living with dementia. Prioritizing a user-testing component with carers increases the likelihood of refining and improving the usability of an intervention that is culturally appropriate, user centered, and lays the foundation for a subsequent RCT to test its effectiveness.

## Appendix

Multimedia Appendix 1 Online surveys completed by user-testing participants using REDCap (Research Electronic Data Capture).

Multimedia Appendix 2 Sequential website tasks completed by user-test participants.

Multimedia Appendix 3 Screenshots of the Draw-Care website showing (1) the language selection page, (2) the log-in page, and (3) a sample of the main home page.

## Abbreviations

eHEALS: eHealth Literacy Scale
GAI: generative artificial intelligence
IDA: instant data analysis
RCT: randomized controlled trial
REDCap: Research Electronic Data Capture
WHO: World Health Organization

## Data Availability

The datasets generated and analyzed during this study are available from the corresponding author on reasonable request.
