# Supplementary material for: Draw-Care, a Co-Designed Multilingual Digital Intervention for Family Carers of People Living With Dementia From Ethnically Diverse Communities: User-Testing Study
Source: JMIR Form Res. 2026 Mar 3;10:e81128. doi: 10.2196/81128 (PMC12996899; doi:10.2196/81128)
Supplement: Multimedia Appendix 1 [file formative_v10i1e81128_app1.docx]

**Appendix 1. Online surveys completed via RedCap by user testing participants (*n*=30)**

1. Sociodemographic questionnaire variables included gender (male/female/prefer not to disclose), year of birth, age (years), marital status (married, single i.e., never married, divorced/separated, widowed), current living circumstances (live alone in my own home, live with others in my home, live with others in their home), an education level (primary school, high school, University, Vocational), current occupation (free text), current work status (full-time, part-time, casual, unemployed, retired, other), country of birth (free text), number of years lived in Australia (free text), residency status (citizen, permanent, temporary), ethnicity or cultural group you identify with (free text), the main language spoken at home (other than English), English proficiency level (none, basic, conversational, professional, native-speaker), religion (free text), number of years as a carer (free text), the relationship of the carer to the person living with dementia (free text).
2. The 8-item e-Health Literacy Scale (eHEALS) was used to assess and measure the carer's perceived knowledge and e-health literacy skills. Each item has five response options ranging from strongly disagree (score 1), disagree (score 2), undecided (score 3), agree (score 4), and strongly agree (score 5) with a possible score range from 8 to 40. A higher score—closer to 40, indicates greater self-perceived skills in finding, evaluating, and using electronic information to make decisions about individual health.

- I know what health resources are available on the internet.
- I know where to find helpful health resources on the internet.
- I know how to find helpful health resources on the internet.
- I know how to use the internet to answer my questions about health.
- I know how to use the health information I find on the internet to help me.
- I have the skills I need to evaluate the health resources I find on the internet.
- I can tell high-quality health resources from low-quality health resources on the internet.
- I feel confident in using information from the Internet to make health decisions.

1. Two questions to evaluate a participant's propensity to the internet and its usefulness and importance for health care decision-making and access to resources.

- How *useful* do you feel the Internet is in helping you make decisions about your health? Response options: *not useful at all; not useful; unsure; useful; very useful.*
- How *important* is it for you to be able to access health resources on the Internet? Response options: *not important at all; not important; unsure; important; very important.*
