# Supplementary material for: Draw-Care, a Co-Designed Multilingual Digital Intervention for Family Carers of People Living With Dementia From Ethnically Diverse Communities: User-Testing Study
Source: JMIR Form Res. 2026 Mar 3;10:e81128. doi: 10.2196/81128 (PMC12996899; doi:10.2196/81128)
Supplement: Multimedia Appendix 2 [file formative_v10i1e81128_app2.docx]

**Appendix 2. Sequential website tasks completed by user test participants.**

To start, the researcher (AT or THD) briefly introduced the Draw-Care web-based intervention. Participants were asked to log in to the website by accessing the link and code provided via email, then follow the website prompts to select their preferred language followed by entering their access code to navigate to the home page.

Then, each participant was asked to complete a set of sequential representative tasks on the website and provide ‘think-aloud’ feedback as they completed these tasks while the researcher took extensive notes. Sessions were not recorded.

1) Locate the menu bar.

2) Identify a short film to watch.

3) Watch the selected film by locating the play button.

4) Locate the accompanying practical 'Tips’ sheet and read it by locating the appropriate button to access and open this information.

5) Participants were asked to identify and locate the virtual ‘Helper’ and the ‘Feedback’ features to complete these tasks and were asked to locate the rating scale and use it to rate the film they selected and watched.
