# Supplementary material for: Draw-Care, a Co-Designed Multilingual Digital Intervention for Family Carers of People Living With Dementia From Ethnically Diverse Communities: User-Testing Study
Source: JMIR Form Res. 2026 Mar 3;10:e81128. doi: 10.2196/81128 (PMC12996899; doi:10.2196/81128)

**Appendix 3: Screenshots showing the Draw-Care website’s 1) language selection page, followed by 2) the login page, and 3) a sample of the main home page.**


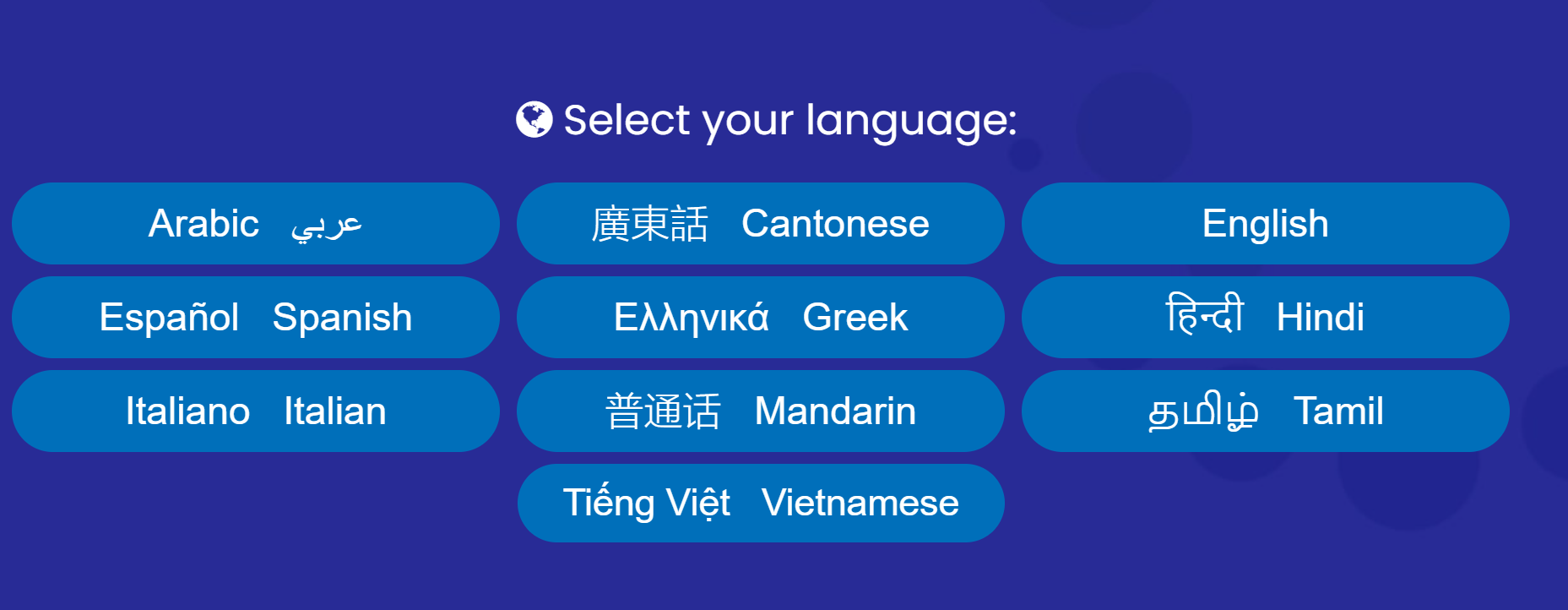


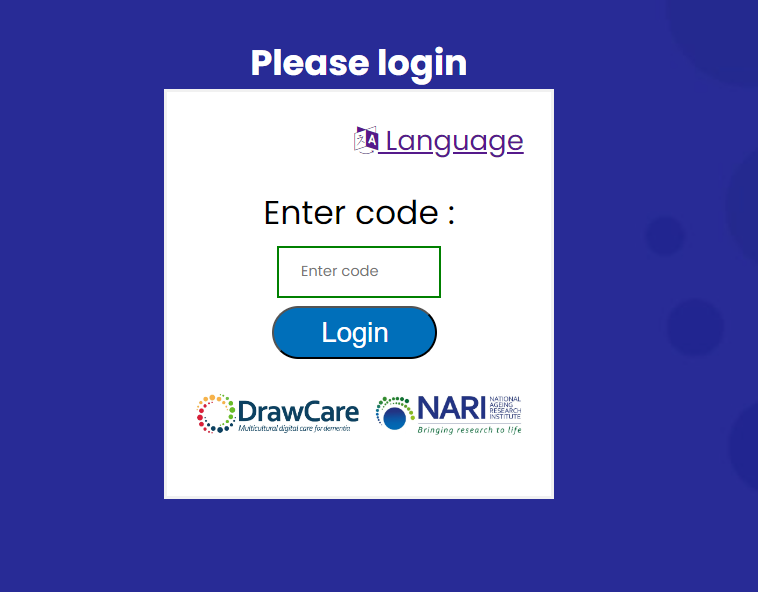

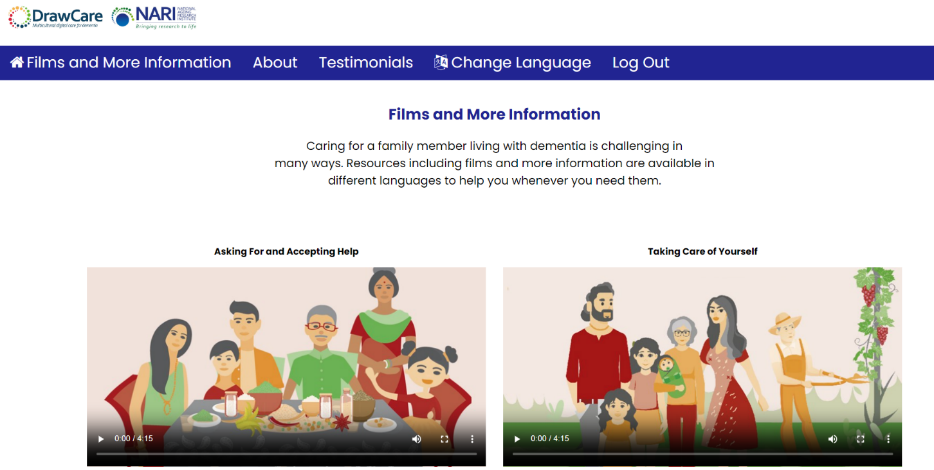

Supplement: Multimedia Appendix 3 [file formative_v10i1e81128_app3.docx]
